# 317. Use cases for rapid antigen-detecting tests for COVID-19 screening and surveillance: a systematic review

**DOI:** 10.1093/ofid/ofac492.395

**Published:** 2022-12-15

**Authors:** Apoorva Anand, Jacob Bigio, Emily MacLean, Talya Underwood, Nitika Pant Pai, Sergio Carmona, Samuel G Schumacher, Amy Toporowski

**Affiliations:** Research Institute of the McGill University Health Centre, India; Research Institute of the McGill University Health Centre; McGill International TB Centre, Montreal, Quebec, Canada; McGill International TB Centre; Department of Epidemiology, Biostatistics and Occupational Health, McGill University, Montreal, Quebec, Canada; Anthos Communications, Northwich, England, United Kingdom; Research Institute of the McGill University Health Centre; Division of Clinical Epidemiology, Department of Medicine, McGill University, Montreal, Quebec, Canada; FIND, Geneva, Geneve, Switzerland; FIND, Geneva, Geneve, Switzerland; FIND, Geneva, Geneve, Switzerland

## Abstract

**Background:**

Testing remains critical to controlling the COVID-19 pandemic. Antigen-detecting rapid diagnostic tests (Ag-RDTs), which can be used at the point of care, have the potential to increase access to COVID-19 testing, particularly in settings with limited laboratory capacity. This systematic review synthesized literature on specific use cases and performance of Ag-RDTs for detecting SARS-CoV-2, for the first comprehensive assessment of Ag-RDT use in real-world settings.

**Methods:**

We searched three databases (PubMed, EMBASE and medRxiv) up to 12 April 2021 for publications on Ag-RDT use for large-scale screening and surveillance of COVID-19, excluding studies of only presumptive COVID-19 patients. We tabulated data on the study setting, populations, type of test, diagnostic performance, and operational findings. We assessed risk of bias using QUADAS-2 and an adapted tool for prevalence studies.

**Results:**

From 4313 citations, 39 studies conducted in asymptomatic and symptomatic individuals were included. Of 39 studies, 37 (94.9%) investigated lateral flow Ag-RDTs and 2 (5.1%) investigated multiplex sandwich chemiluminescent enzyme immunoassay Ag-RDTs. Six categories of testing initiatives were identified: mass screening (n=13), targeted screening (n=11), healthcare entry testing (n=6), at-home testing (n=4), surveillance (n=4) and prevalence survey (n=1). Sensitivity and specificity values by testing category are shown in the table. Ag-RDTs were noted as convenient, easy-to-use, and low cost, with a rapid turnaround time and high user acceptability. Risk of bias was generally low or unclear across studies.

**Figure:**
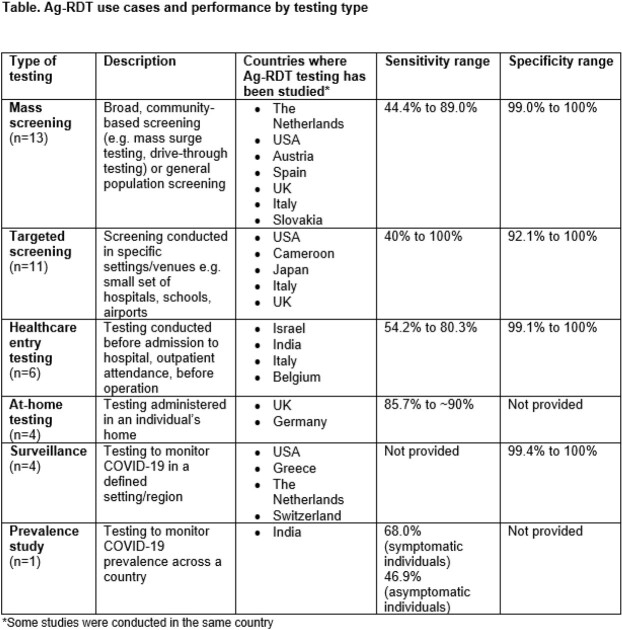

**Conclusion:**

During the first year of the COVID-19 pandemic, Ag-RDTs were used across a wide range of real-world settings for screening and surveillance of COVID-19 in both symptomatic and asymptomatic individuals. Ag-RDTs were fast and simple to run, but due to their often low sensitivity, careful consideration must be given to their implementation and interpretation. Ag-RDTs have subsequently been rolled out more broadly and recommended for COVID-19 self-testing.

**Disclosures:**

**Talya Underwood, MPhil**, Oncotherapeutics: Medical writing.

